# A Presentation of Meige Syndrome With Associated Upper Motor Neuron Symptoms

**DOI:** 10.7759/cureus.60101

**Published:** 2024-05-11

**Authors:** Jeffrey Valencia Uribe, Nazia Hossain, Hassnain Rizvi, Mohammed Qureshi

**Affiliations:** 1 Internal Medicine, Memorial Healthcare System, Pembroke Pines, USA; 2 Neurology, Memorial Healthcare System, Pembroke Pines, USA

**Keywords:** cervical dystonia, upper motor neuron signs, oromandibular dystonia, blepharospasm, meige syndrome

## Abstract

Meige syndrome (MS) is a cranial dystonia that involves blepharospasm and oromandibular dystonia. It can also evolve to include other adjacent muscle groups in the cervical region. It typically presents in middle-aged females, and while the disorder is relatively uncommon, its exact prevalence varies. Diagnosis is typically made with a thorough history and physical and workup to rule out other causes. Treatment options include medical management with gamma-aminobutyric acid (GABA) antagonists, dopamine antagonists, and anticholinergics for short-term management. Long-term treatment options are Botox and deep brain stimulation. This case report presents a 56-year-old female with a complex presentation of MS; the patient's symptoms progressed from isolated blepharospasms to involve orofacial and cervical musculature. A distinctive aspect of this case was the simultaneous presence of upper motor neuron (UMN) signs in the patient alongside acute to subacute compression fractures of the superior endplate of C7 and T3, as revealed by cervical spine imaging. Treatment with clonazepam led to significant symptomatic improvement, highlighting the importance of a multimodal approach in managing MS. This case underscores the need for careful clinical evaluation, collaboration with movement disorder specialists, and ongoing research efforts to enhance understanding and treatment of MS.

## Introduction

Meige syndrome (MS) is a form of segmental craniocervical dystonia characterized by the co-occurrence of blepharospasm (involuntary eye blinking) and oromandibular dystonia (involuntary movements affecting the lower face and jaw) [1]. Henry Meige, a pioneering French neurologist in the early 20th century, meticulously documented this amalgamation of symptoms, thereby establishing its recognition within neurological practice [2]. Symptoms typically initiate with the involvement of the orbicularis oculi muscles, ranging from heightened blinking to eventual eye closure difficulties. As the condition progresses, it commonly affects the lower face and jaw muscles, leading to manifestations such as jaw clenching, grimacing, or jaw thrusting [3]. Spread to neighboring muscle groups, including the sternocleidomastoid, trapezius, and other cervical muscles, is also prevalent, with the majority occurring within the initial three to five years of onset [4]. 

A comprehensive study conducted by Berman et al. in 2020, encompassing 487 participants with adult-onset focal dystonia (of which 50% originated from blepharospasm), highlighted that the oromandibular region (42.2%) and neck (22.4%) were the most commonly affected regions during initial spread [5]. MS exhibits a higher prevalence in females and typically emerges between the ages of 30 and 70, with a median onset age of 55.7 years [6]. Hence, clinicians should maintain a heightened clinical suspicion for MS in middle-aged females, presenting with new-onset blepharospasm. Despite ongoing research efforts, the precise etiology of primary MS remains elusive, with evidence suggesting a multifaceted origin, involving genetic predisposition, environmental factors, and neurochemical imbalances [7]. Various hypotheses have been proposed, including aberrant control of cranial nuclei, dopaminergic hyperactivity, and reduced grey matter in specific brain regions. However, a consensus on the etiology has yet to be reached [6,8]. Our case report endeavors to illustrate a clinical presentation of MS and to augment clinicians' awareness regarding focal dystonic movement disorders and their therapeutic management.

## Case presentation

A 56-year-old female presented with a constellation of involuntary movements, including bilateral eyelid and neck contractions, accompanied by tremors, dysphagia, and dysarthria. Her medical history included hyperlipidemia and depression, the duration of which was unknown. Upon admission, she was taking 10 mg of atorvastatin and 10 mg of paroxetine daily. The onset of symptoms commenced with blepharospasms, escalating to involve orofacial and cervical muscles over the next four months. Orofacial symptoms included involuntary yawning and chewing motions leading to dysphagia and dysarthria. These episodes of involuntary muscle contractions occurred approximately every few minutes, often followed by suboccipital headaches radiating to the cervical spine. Notably, there were no recent fevers, chills, weight loss, vision changes, or trauma reported by the patient.

Upon examination, recurrent bilateral blepharospasms were observed, alongside an episode of right-sided cervical spasm. Increased muscle tone and hyperreflexia were evident in bilateral upper and lower extremities. Additionally, the patient exhibited several left upper extremity symptoms, including abduction of the left thumb, intermittent tremors in the left arm, and a positive Hoffman's sign on the left side. While ambulating, the patient displayed a broad-based gait, leaning notably toward the right, accompanied by tremors in the right lower extremity.

**Figure 1:**
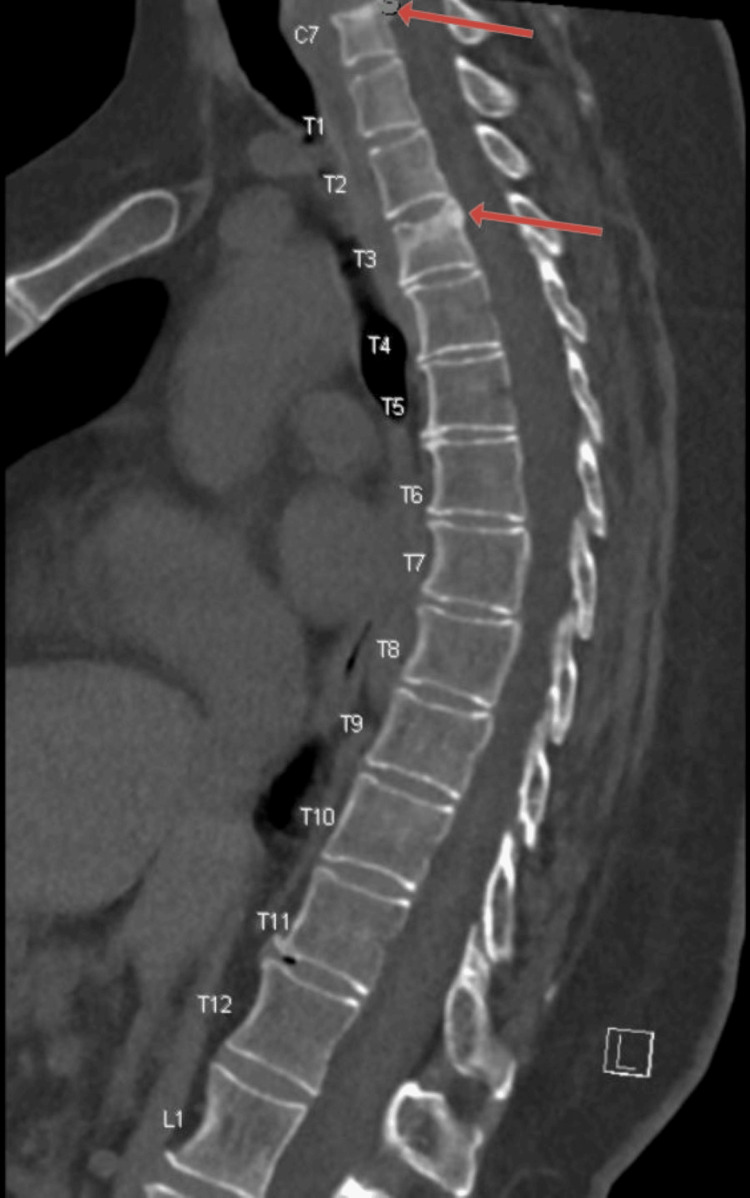
CT thoracic spine without IV contrast Demonstrating a subacute to a chronic fracture involving the superior endplate of C7 and an acute to subacute superior endplate fracture involving T3 (red arrows)

The laboratory findings revealed a low thyroid-stimulating hormone (TSH) (0.289), and a slight decrease in free T4 (0.8). Other investigations were negative for vitamin B12 deficiency, inflammatory markers, autoimmune processes, syphilis, HIV, copper, ceruloplasmin, or any other metabolic derangements. The EEG results were essentially normal, without any potentially epileptogenic abnormalities. Imaging studies, including computed tomography (CT) brain, CT arteriogram brain, magnetic resonance imaging (MRI) brain, and CT lumbar (L) spine were unremarkable. However, an MRI of the cervical spine revealed acute to subacute compression fractures of the superior endplate of cervical (C) 7 and thoracic (T) 3, without cord signal abnormality. These abnormalities were better visualized on the CT thoracic spine without IV contrast (Figure 1), demonstrating superior endplate fractures involving T3 and C7.

The patient's clinical presentation featured blepharospasms initially, with subsequent progression to involve orofacial and cervical muscles, resulting in considerable discomfort. The examination revealed recurrent bilateral blepharospasms, right-sided cervical spasms, and various upper extremity neurological symptoms. These findings raised suspicion of MS, with no other significant findings in neurometabolic disturbances or neurological imaging to suggest an alternative diagnosis. The treatment involved the administration of clonazepam at a dosage of 0.5 mg every eight hours, resulting in a significant reduction in both dystonic and bulbar symptoms within the initial 24-hour period. However, upper motor neuron (UMN) signs persisted. Neurosurgical intervention was not required for the compression fractures; instead, conservative management was initiated, including the placement of a cervical collar for a duration of six weeks. The patient was discharged with a prescription for clonazepam at a dosage of 0.5 mg twice daily as needed. It was advised to follow up with a movement disorder neurologist for further testing.

## Discussion

This case presents a distinctive manifestation of focal dystonic movement disorder accompanied by UMN signs. Among the potential differential diagnoses considered were stroke, tardive dyskinesia (TD), cervical myelopathy, and MS. The stroke was eliminated as brain imaging revealed no abnormalities, and symptoms were atypical. The absence of medication exposure associated with TD made it an improbable diagnosis. 

Diagnosing MS relies on clinical observations, notably the presence of both blepharospasms and oromandibular dystonia, coupled with the exclusion of other underlying neurological conditions [4]. Electromyography (EMG) and neuroimaging, such as MRI, are pivotal in ruling out other movement disorders and structural abnormalities. Typically, initial symptoms manifest as essential blepharospasm or oromandibular spasms, gradually spreading bilaterally and involving additional muscle groups. In the case at hand, symptoms initiated with blepharospasms progressed to encompass orofacial and cervical musculature over the ensuing months. Notably, the patient exhibited UMN lesion signs, including a positive Hoffman's sign on the left side, heightened muscle tone, and hyperreflexia in bilateral upper and lower extremities. Although neuroimaging yielded negative results, the MRI/CT of the cervical spine uncovered acute to subacute compression fractures of the superior endplate of C7 and T3, devoid of cord signal abnormalities.

While the association between MS and UMN lesions or spinal compression fractures remains elusive, certain reports suggest a potential link between compression fractures and generalized dystonia, particularly retrocollis prevalence. This hints at a possible interplay between underlying bone density irregularities and the strain induced by spasmodic torticollis [9]. Despite several case series highlighting connections between dystonic disorders and cervical myelopathy, the absence of radiographic anomalies within the spinal cord challenges conventional understanding [9,10].

The pathophysiology and neural mechanisms underlying MS remain elusive; however, a study employing functional MRI and EMG in experimental models has shed light on potential factors [11]. Specifically, patients with blepharospasm exhibit heightened activation in the caudate nucleus and sensorimotor cortices during the trigeminal blink reflex, indicating a possible loss of inhibition within these regions. It is also theorized that cortical preparatory irregularities may initiate alterations within the premotor and motor cortex, which are involved in oromandibular movement [12]. Moreover, voxel-based morphometry analysis of MRI scans in a cross-sectional study revealed functional abnormalities rather than structural changes in the basal ganglia and motor cortex in MS patients [8]. Notably, a decrease in gray matter volume in the precuneus, known for its connectivity with the prefrontal lobe and basal ganglia nuclei, suggests a pivotal role of the precuneus in MS pathogenesis. Additionally, MS has been linked to various movement disorders, including Parkinson's disease, atypical parkinsonism, and Wilson's disease [7]. Moreover, similar dystonic symptoms observed in patients with ocular palatal tremors point toward pseudohypertrophy of the inferior olive, associated with focal lesions in the Guillain-Mollaret triangle (MGT) [13]. The MGT encompasses a network of neurons in the brainstem crucial for motor control and coordination, potentially implicated in dystonic movement disorders such as MS. However, despite these associations, our patient's clinical presentation and findings did not align with these conditions, suggesting a unique manifestation of MS.

The management of MS focuses on addressing blepharospasm and oromandibular dystonia through a combination of medications, botulinum toxin injections, and deep brain stimulation (DBS) [14]. Botulinum toxin injections, particularly onabotulinumtoxin A (OnaBoNT/A), are considered first-line treatment for various dystonias, as evidenced by a systematic review of randomized masked trials [15]. A notable trial for blepharospasms demonstrated a reduction of 38.9% in symptoms with 25 U of Botox (AbbVie Inc., North Chicago, USA) per eye initially, followed by 50 U per eye if necessary after a month, with effects lasting around three months [16]. Adverse effects may include blurred vision, ptosis, and periorbital hematoma. Additionally, studies have explored Botox doses ranging from 70 to 240 U for cervical dystonia, with notable improvements in combined oromandibular-cervical dystonia, albeit with dysphagia as a common adverse effect [15,16].

Pharmaceutical therapy utilizing anticholinergics, benzodiazepines, and baclofen can offer initial relief and serve as interim measures between Botox injections [14]. However, their efficacy varies, often accompanied by multiple systemic side effects. While DBS hasn't been extensively explored in MS, it's emerged as a promising alternative for treating dystonia that doesn't respond to botulinum toxin injections [14]. In a study involving six MS patients who underwent bilateral stereotactic implantation of DBS leads into the sensorimotor globus pallidus internus (GPi), there was a remarkable 72% improvement on the dystonia rating scale observed at the six-month mark post-operation [17]. Moreover, a retrospective study evaluating clinical outcomes in MS patients treated with bilateral GPi DBS showed similarly encouraging improvements sustained over long-term follow-ups exceeding one year [18]. In our case, we initiated treatment with a benzodiazepine, resulting in significant symptomatic improvement. The patient was recommended to follow up with a movement disorder specialist for botulinum toxin injections. 

The primary limitations in this case revolved around the diagnostic work-up process, which was inevitable due to the constraints inherent in an inpatient setting. While EMG studies are recommended for a comprehensive evaluation of MS, conducting them in an inpatient context proved impractical. The absence of EMG evaluation can impede both diagnosis and treatment planning, as EMG studies are invaluable in distinguishing MS from other movement disorders associated with dystonia or Parkinson's disease. Furthermore, EMG can elucidate the specific muscles involved and their level of hyperactivity, aiding in selecting appropriate treatment modalities [19,20]. The longitudinal EMG assessments also offer insights into treatment response by tracking changes in muscle activity over time.

## Conclusions

This case report aims to illuminate a distinctive manifestation of MS and elevate clinicians' vigilance regarding focal dystonic movement disorders and their management. The patient's evolving clinical presentation, initially marked by blepharospasms and later involving orofacial and cervical musculature, epitomized the classical features of MS. Nonetheless, the concurrent manifestation of UMN signs and the identification of acute to subacute compression fractures of the superior endplate of C7 and T3 introduced nuanced complexities into the diagnostic trajectory, rendering our case uniquely challenging. It is imperative to discern the clinical presentation accurately and differentiate focal dystonic movement disorders from psychogenic etiologies. Therapeutically, addressing MS often necessitates a multimodal approach, encompassing pharmacotherapy with benzodiazepines, anticholinergics, or baclofen, coupled with botulinum toxin injections, and, in recalcitrant cases, DBS can be considered. Looking ahead, sustained longitudinal monitoring and collaborative engagement with movement disorder specialists remain pivotal for optimizing treatment efficacy and mitigating potential complications. Furthermore, continued research endeavors to elucidate the underlying pathophysiological mechanisms and explore innovative therapeutic modalities are imperative to augment our comprehension and management of this intricate clinical entity.
